# Variant analysis of *PEX11B* gene from a family with peroxisome biogenesis disorder 14B by whole exome sequencing

**DOI:** 10.1002/mgg3.1042

**Published:** 2019-11-13

**Authors:** Yuan Tian, Linlin Zhang, Ying Li, Jinshuang Gao, Haiyang Yu, Yaqing Guo, Liting Jia

**Affiliations:** ^1^ Department of Clinical Laboratory The Third Affiliated Hospital of Zhengzhou University Zhengzhou China; ^2^ Screening Center The Third Affiliated Hospital of Zhengzhou University Zhengzhou China

**Keywords:** peroxisome biogenesis disorder 14B, *PEX11B* gene, variant analysis, whole exome sequencing

## Abstract

**Background:**

Peroxisome biogenesis disorder 14B (PBD14B) is an autosomal recessive peroxisome biogenesis disorder characterized clinically by mild intellectual disability, congenital cataracts, progressive hearing loss, and polyneuropathy peroxisome biogenesis disorders are genetically heterogeneous group of disorders caused by biallelic mutations in peroxin (*PEX*) genes.

**Methodology/Laboratory Examination:**

DNA of the family was extracted and sequenced by whole exome sequencing. The results were validated with Sanger sequencing analyzed with Bioinformatics software.

**Results:**

Sequencing result showed that the patient has carried a homozygous variant of c.277C>T of the *PEX11B* gene. The patient's brother has carried a homozygous variant of c.277C>T of the *PEX11B* gene and their variants of c.277C>T of the *PEX11B* gene were inherited, respectively, from his mother and father.

**Discussion and Conclusion:**

The homozygous variant of c.277C>T of the *PEX11B* gene probably underlie the disease in this child and her brother.

## Graphical abstract

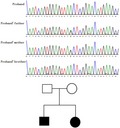

## 1 INTRODUCTION

Peroxisome biogenesis disorder 14B (PBD14B) is an autosomal recessive peroxisome biogenesis disorder characterized clinically by mild intellectual disability, congenital cataracts, progressive hearing loss, and polyneuropathy (Taylor et al., 2017). Eukaryotic peroxisomes are single‐membrane bound organelles harboring enzymes that operate in metabolic pathways critical for normal human development and health and are present in almost all cells (Li & Gould, 2003; Wanders & Waterham, 2006).

Human peroxisomal disease is caused by mutations in the genes encoding two classes of protein: single enzyme or metabolite transporter defects known as peroxisomal enzyme deficiencies (PEDs), the clinical and biochemical effects of which are dependent on the specific function of the affected protein; or peroxisome biogenesis disorders (PBDs) that result from a universal abnormality of peroxisome function due to a specific defect in one of the processes crucial for the assembly or maintenance of peroxisomes (Faust, Banka, Siriratsivawong, Ng, & Wikander, 2005; Steinberg et al., 2006; Weller, Gould, & Valle, 2003).Peroxisome biogenesis disorders are a genetically heterogeneous group of disorders caused by biallelic mutations in peroxin (*PEX*) genes (Abe & Fujiki, 1998). Reports on peroxisome biosynthesis disorders caused by *PEX11B* gene mutations are relatively rare so far (Ebberink et al., 2012).

We performed a gene mutation analysis on a patient with PBD14B by whole exome sequencing technology, providing further diagnosis and treatment basis for clinical diagnosis and genetic counseling.

## 2 CASE PRESENTATION

The proband, female, 9 years old, who was admitted to the hospital with "fever" as the main complaint. Physical examination: bilateral nystagmus, congenital cataract with myopia, strabismus, high muscle tone, and mental retardation. The proband is the first child of his nonclose relatives. There is a younger brother in this family. Her brother has similar symptoms, but the symptoms were slightly mild. The clinical manifestations are occasional tremors in the bilateral eyeballs, mild cataracts with strabismus amyopia, meanwhile the muscle tension was slightly higher. Blood routine of the proband: white blood cells (WBC): 17.22 × 10^9^/L (normal reference value: 3.5–12 × 10^9^/L), hemoglobin (Hb): 83.00 g/L (normal reference value 110–170 g/L). The parents have no relevant symptoms and the parents are not close relatives.

## 3 DNA EXTRACTION

The proband, the proband's brother, and their parents' peripheral blood 3 ml were collected into the EDTA anticoagulation tube, and the genomic DNA was extracted using QIAGEN 69504 blood and tissue DNA extraction kit.

## 4 WHOLE EXOME SEQUENCING

The genomic DNA of the peripheral blood of the proband and her parents was broken into random fragments and purified, and whole genome exome capture was performed for sequencing library preparation. The Illumina Hiseq XTen sequencer was used to perform double‐ended high‐throughput sequencing with a length of 150 bp. The raw data obtained by the sequencing were quality‐controlled, and the basic data were analyzed and filtered to remove the linker sequence and repeat sequence. Data were analyzed using the Anno variant site detection system and the Exomiser, Phenolyzer variant site annotation interpretation system.

## 5 BIOINFORMATICS ANALYSIS

The mutation frequency was analyzed using the unique database of the Berry Gene Chinese population “Shenzhou Genome Database” and the reference population of 1,000 human genome (1000G), dbSNP, ExAC, and gnomAD databases, and the common variation of the allele population frequency greater than 0.005 was filtered out. Harmful predictions using the prediction software Polyphen‐2, SIFT, Mutation Taster, CADD, etc. The OMIM, Clinvar, and HGMD databases were reviewed and the mutations were graded in conjunction with the American Society of Medical Genetics and Genomics (ACMG) grading standards to comprehensively determine the pathogenicity of the variation.

## 6 SANGER SEQUENCING

Genetic modification of the mutations was performed on the proband, the proband's younger brother and their parents. According to the exon of the mutation site, the primers needed for sequencing the synthetic DNA fragments were designed, and the DNA of the proband, the proband's younger brother and the parents were PCR‐amplified. Method of sequencing was performed by Sanger sequencing (Table 1), and the sequencing results were compared with the results of whole exon sequencing.

**Table 1:** *PEX11B* gene Sanger sequencing primer sequence

Primer name	Forward primer sequence (5′‐3′)	Reverse primer sequence (5′‐3′)	Annealing temperature (°C)	Product length (bp)
PEX11B c.277C>T	TCTCCACAAGTTCTACGCCTG	CTAAGTGATTGGAAGCCAAGTG	60	351

## 7 BIOCHEMICAL ANALYSIS

Very‐long‐chain fatty acids, Prolines, Phytanic acid, Bile acid intermediates and plasmalogens measurements in plasma from peripheral blood samples were used to measure peroxisomal biochemical parameters in this family by liquid chromatography/tandem mass spectrometry (Morin‐Rivron, Christinat, & Masoodi, 2017).

## 8 RESULTS

**Figure 1:**
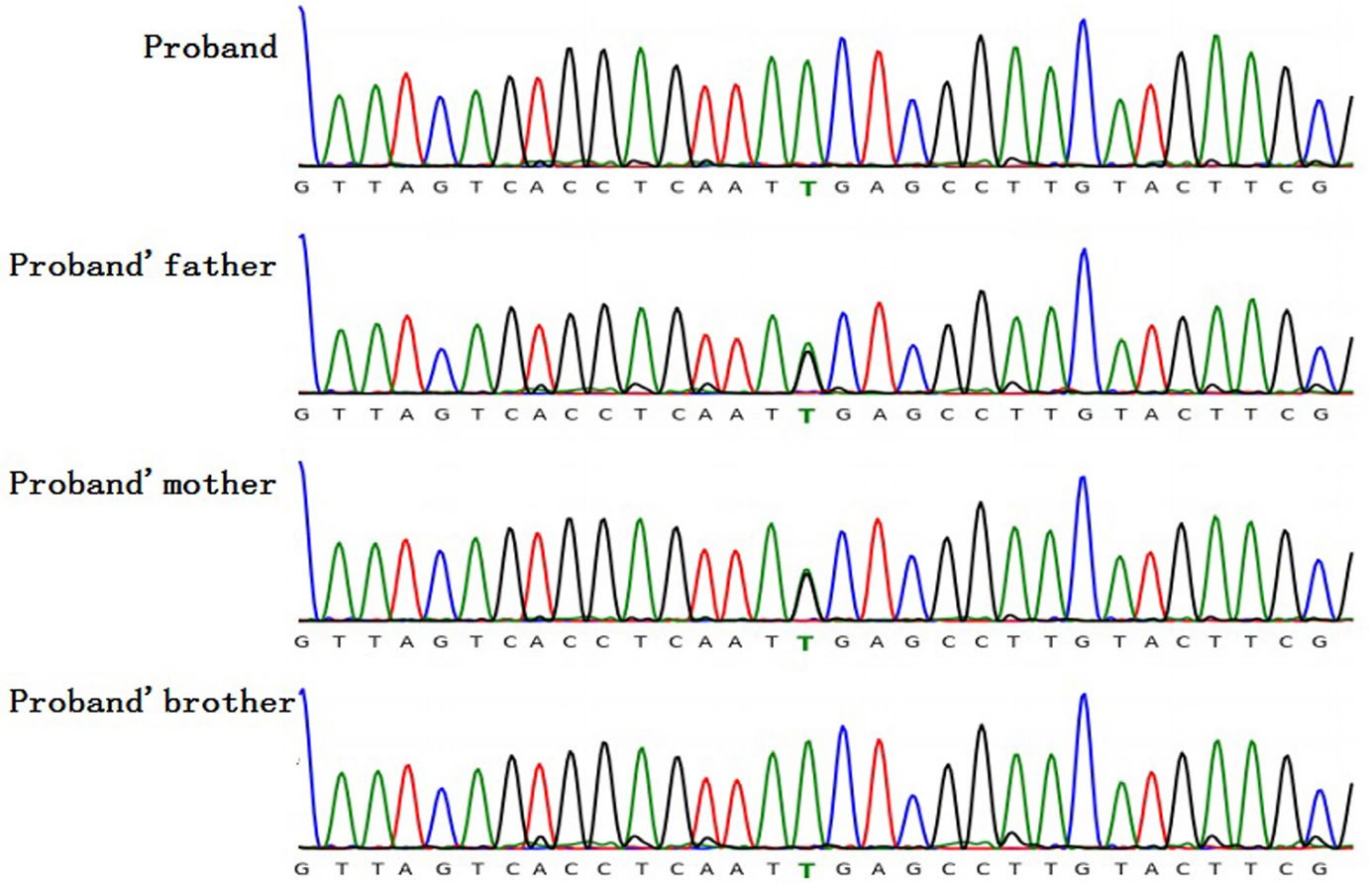
The Sanger sequencing result of proband, proband's father, proband's mother and proband's brother

By calculating the target gene coverage with a sequencing depth of 20 × or above, the proband was 97.64%, the father was 97.08%, and the mother was 96.47%. Therefore, the data quality of the family was in compliance with the requirements. By detecting and screening the data in the mutation list, a homozygous candidate mutation site c.277C>T (p.R93X) homozygous variation was found in the *PEX11B* gene (NM_003846.2). Through the results of the family site verification, it was found that both the father and the mother carried a *PEX11B* gene c.277C>T (p.R93X) heterozygous variation, and the younger brother had the same *PEX11B* gene c.277C>T ((the same as the child) p.R93X) homozygous variation (Figure 1).

According to the American Society of Medical Genetics and Genomics (ACMG) guidelines (Poll‐The et al., 1987; Richards et al., 2015), the c.277C>T mutation of the *PEX11B* gene is suggested to be a pathogenic mutation site (Table 2). Mutations in the *PEX11B* gene lead to peroxisome biosynthesis disorders of type 14B in autosomal recessive disorders (Ebberink et al., 2012). Peroxisome biosynthesis disorder 14B is mainly characterized by congenital cataract, nystagmus, mild intellectual disability, and muscle weakness (Ebberink et al., 2012). The disease characteristics are consistent with the phenotype of the case.

**Table 2:** Evidence of pathogenicity of *PEX11B* gene

Pathogenicity	Evidence of pathogenicity
pvs1	This mutation mutates the corresponding codon to a stop codon, resulting in a change in protein function
pm2	The mutation was not found in the Berry Gene Chinese population‐specific database "Shenzhou Genome Database" and the reference population Thousand Human Genome (1000G). Both in the human exon database (ExAC) and the population genome mutation frequency database (gnomAD) are 0%
pp3	Conservative prediction by CADD and GERP showed that the site was evolutionarily conserved and had potential functional effects. The protein function was predicted by DANN and the results were shown to be harmful

The concentrations of very long chain fatty acids, bile acid intermediates (Pipecolic acid), plasmalogens and phytanic acid were measured by biochemical analysis of probands in plasma. It was found that the above biochemical indicators were normal in the plasma of the proband (Table 3).

**Table 3:** Standard of biochemical indicators measured in plasma of proband

Biochemical indicators	Proband	Control
Very‐long‐chain fatty acids		
C26:0	0.78	0.45–1.32
C24:0/C22:0	0.91	0.57–0.92
C26:0/C22:0	0.02	0.003–0.02
Phytanic acid	2.2 μg/ml	0–3.1 μg/ml
Pipecolic acid	3.8 μmol/L	0.1−7 μmol/L
Plasmalogens		
C16:DMA/C16:0	0.098	0.079–0.128
C18:DMA/C18:0	0.207	0.199–0.284

## 9 DISCUSSION

PBD14B is an autosomal recessive peroxisome biosynthesis disorder with clinical manifestations of mild mental retardation, congenital cataract, progressive hearing loss, and multiple neuropathy (Ebberink et al., 2012; Kelley et al., 1986; Opalinski et al., 2012). It is currently known that PBD14B is caused by a mutation in the *PEX11B* gene on chromosome 1q21. The *PEX11B* gene consists of four exons and expresses the human peroxisome membrane protein 11‐β (PEX11‐β). PEX11‐β has two transmembrane domains that can be predicted (Gillespie et al., 2016; Li et al., 2002), one domain at the C‐terminus and the other at about 100 amino acid positions from the N‐terminus (Bonekamp et al., 2013; Schrader, Almeida, & Grille, 2012). Schrader et al. (1998). have found in vitro cell experiments that PEX11‐β can be separated from other peroxisome membrane proteins by peroxisome extension and PEX11‐β without extracellular stimulation. The manner of peroxisome cleavage induces peroxisome proliferation in human cells.

Li et al. (2002) used gene targeting technology to generate mice lacking the *PEX11B* gene. After histological analysis, mice lacking the *PEX11B* gene were found to exhibit severe neurological dysfunction, craniofacial malformations, and liver dysfunction. There are features such as neuronal migration defects and developmental delay. To date, only a limited number of peroxisome biosynthesis disorders caused by *PEX11B* gene mutations have been discovered clinically. In 2012, Ebberink et al. (2012) found a 26‐year‐old patient with peroxisome biogenesis disorder 14B (PBD14B) caused by homozygous variation of the *PEX11B* gene c.64C>T. Taylor et al. (2017), found that the underlying causes of disease in three families were *PEX11B* c.235C>T p.(Arg79Ter) homozygous; *PEX11B* c.136C>T p.(Arg46Ter) homozygous; *PEX11B* c.595C>T p.(Arg199Ter) heterozygous, PEX11B ex1‐3 del heterozygous.

**Figure 2:**
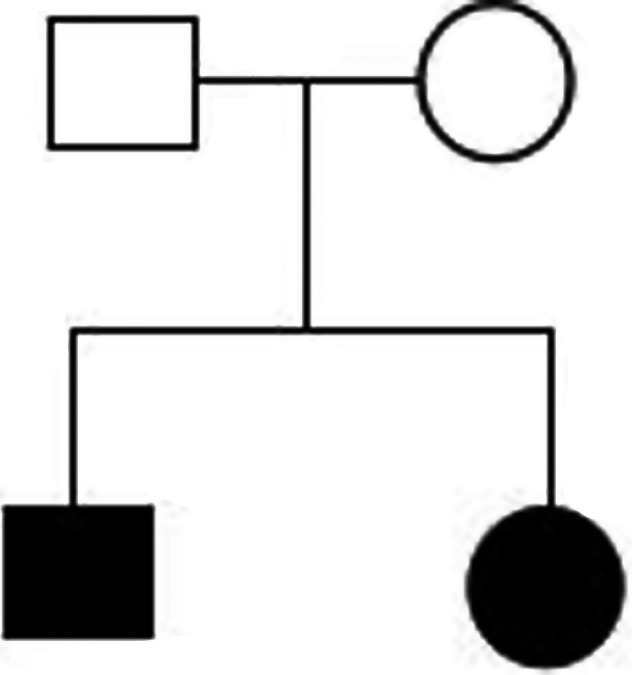
The situation of the family

In our case, the p.R93X homozygous variant present in the proband is a change in codon 93 of exon 3, causing the corresponding codon to be mutated to a stop codon, resulting in a truncated protein resulting in a protein polypeptide chain. The synthesis is terminated prematurely, which in turn affects protein function. In this case, the proband showed a bilateral nystagmus, congenital cataract with myopia, strabismus, high muscle tone, mental retardation, consistent with the PBD14B phenotype. The younger brother of the proband had similar clinical manifestations. Through the family results, the homozygous mutation of the *PEX11B* gene c.277C>T site was found to be consistent with the proband. The mutation site was sequenced by Sanger sequencing and found that both of parents carried the *PEX11B* gene. c.277C>T site heterozygous mutation, and both of parents have normal phenotype, the family is in line with Mendelian inheritance law (Figure 2). Therefore, the homozygous variation of the *PEX11B* gene c.277C>T may be the cause of the disease caused by the proband and her brother. In this family, the biochemical indicators of the proband were normal, which is consistent with previous studies (Ebberink et al., 2012).

This family is the first genetically transmitted family to be reported in China to cause peroxisome biosynthesis disorders due to *PEX11B* gene mutations. This discovery broadens the pathogenic profile of the disease‐causing gene and provides clinical diagnosis and genetic counseling. More extensive basis for diagnosis and treatment.

## CONFLICT OF INTEREST

We declare that we do not have any commercial or associative interest that represents a conflict of interest in connection with the work submitted.

## AUTHORS CONTRIBUTION

Tian Yuan participated in the overall design. Li Ying participated in the follow‐up. Gao Jinshuang participated in the experimental data analysis. Yu Haiyang and Guo Yaqing participated in the experimental operation. Jia Liting and Zhang Linlin are corresponding authors of this manuscript.
